# Degradation kinetics and physiological studies of organophosphates degrading microorganisms for soil bioremediation

**DOI:** 10.1007/s44154-023-00138-6

**Published:** 2024-02-06

**Authors:** J. M. Kilonzi, S. Otieno

**Affiliations:** Kenya Agricultural and Livestock Research Organization Tigoni, Limuru, P.O BOX 338-0217 Kenya

**Keywords:** Microbes isolation, Agrochemicals, Degradation, Strains, Organophosphate

## Abstract

Organophosphate compounds are widely used in agricultural activities to optimize food production. Contamination of field soil by these compounds may result in detrimental effects on soil biota. The aim of the present study was to isolate microorganisms from field soils and evaluate the strains on ability to degrade organophosphates as single isolate and as a consortium. Isolated strains were identified using both biochemical and molecular techniques. Results revealed that, out of the 46 isolated strains, three isolates herein referred to as S6, S36 and S37 showed an average diazinon degradation rate of 76.4%, 76.7% and 76.8% respectively, of the initial dose (50 ppm) within 11 days of incubation in mineral medium. Notably, isolates S36 and S37 were more effective than S6 in degrading diazinon by 40% in soil aliquot after 11 days and therefore were evaluated on biochemical reactions and molecular identification. The isolates showed variable biochemical characteristics. However, both isolates possessed catalase enzyme, but lacked oxidase enzyme. Molecular characterization showed that, the closest species for S36 and S37 were *Priestia megaterium* and *P. arybattia*, respectively, based on 16S rRNA gene similarity (> 99%). Combination of the strains increased diazinon degradation ability by 45% compared to single strain treatment. Chlorpyrifos was the most highly degraded organophosphate, compared to phorate and cadusafos. Therefore it is expected that the pesticide-degrading bacteria could be a solution to soil health improvement and contribution to the production of safe agricultural products.

## Introduction

As a result of modern farming practices, the levels of organic compounds in soil have increased tremendously over the past decades. Among the most highly utilized and detected organic compounds in field soil are the pesticides as integral component of agriculture (Chowdhury et al. 2014). Agrochemicals have being envisaged as one of the key tools for increasing agricultural intensification in producing food and feed globally (Carvalho 2017). However, the strategy has often raised human health, environment and soil degradation concerns ensuing the destruction of the natural self-regulating ecosystem (Ahmed et al. 2021).

One of the highly utilized agrochemicals in farming are the organophosphates (OPs) pesticides, which occupies a significant proportion of the chemical market share (Market Data Forecast 2020). This is attributed to their profound efficacies in managing crop pests among farmers (Arjmandi et al. 2010). Yet, accumulation of these OPs have been apparent in crop fields at unnaturally high concentrations predisposing crop and crop products to residue accumulation (Kang et al. 2015). In addition, the chemical class has been reported as the most toxic substance and habitually associated with endocrine disruption (Aguilar-Garduño et al. 2013), cancer (Catherine et al. 2016) and hypospadias (Michalis et al. 2014), nervous disorders (Lee et al. 2021) following exposure. Indeed, diazinon is among the most detected organophosphate in crop products despite its ban in some countries (Sapbamrer and Hongsibsong 2014). Diazinon residue in crops above allowable limit (0.01 mg kg^−1^ body weight) have been reported in a number of developed countries (Rahimi et al. 2015). Moreover, extensive application of diazinon in field crops can result in accumulation of the chemical in soil and could be absorbed by growing crop during growing period (Wang et al. 2019, b).

The fate of diazinon in the environment is apparent and includes oxidative decomposition to diazoxon, which rapidly hydrolyze to oxypyrimidine; a more mobile and persistent molecule in the environment than the parent material. The chemical may also be volatilized, absorbed by plants or undergo microbial breakdown (Harper et al. 2009). The hazardous aspects of diazinon and diazoxon to human include; endocrine disruption (ElMazoudy and Attia 2012), nervous disorders (Lee et al. 2021), and liver and kidney damage (Cakici and Akat 2013) and potential to impact on ecosystem balance.

Currently, there are limited approaches in existence for soil revitalization and restoration of soil biological functions. Among the possible mechanisms, is natural degradation processes that include; de-chlorination, cleavage, oxidation and reduction reactions by promiscuous enzymes (Arora and Bae 2014). Weather conditions including light, moisture, heat and humidity, also contribute to pesticides and other xenobiotics degradation or even breaking the chemicals to metabolites that may be less toxic or more harmful to biodiversity and human (Sibanda et al. 2011). Analogous to this, microbes involved in pesticides biodegradation under natural ecosystem reduce substantially, the possibilities of agrochemicals persistence in a contaminated soil (Morales et al. 2021). Under this phenomenon, chemicals and their intermediates are either detoxified or used as a new source of nutritional by the microorganisms (Hayatsu et al. 2000). However, the efficacy of the degradation process, is influenced by physio-chemical properties and residue levels of the chemicals, and duration in which the microbes exposed (Kumar et al. 2017). Thus, optimizing microbes’ bioremediation activities under in situ conditions to provide adequate exposure time for mineralizing of the agrochemicals could be a sustainable approach in revitalization of degraded soil.

Previous studies have revealed successes in bioremediation using microbiomes that form syntrophic associations. The potential of the microorganisms to break down the chemicals has been hypothesized as simple to use, low cost and eco-friendliness (Ibrahim and Essa 2010). In this regard, microorganisms that have metabolic pathway adapted to a wide range of ecological conditions and ability to degrade a number of organophosphates degradation has attracted scientific interests. For instance, Tanmaya et al. (2019) showed that, *Ochrabactrum* species degraded chlorpyrifos effectively within 28 days. Equally, Amani et al. (2019) and Morales et al. (2021) showed that *Pseudomonas* and *Acinetobacter* species degraded up to 80% of the original concentration of both chlorpyrifos and diazinon separately in agricultural soil. The ability of fungi including mycorrhizae to biodegrade pollutants by has also been investigated (Pathak et al. 2017). However, bacteria has gained more study interests over fungi on bioremediation ability due to their higher multiplication rate attribute than fungi (El-Ghany and Ahmed 2016). Unfortunately, bacteria may lose their biodegradation capacity over time as a result of confounding effects, including environmental conditions such as pH, temperature and chemical concentration gradient (Yadav et al. 2016) unlike fungi.

The need to explore for more efficacious microbes remain eminent to enhance tolerance to increased agrochemical residue dosages and climate change impacts. This remain a major challenge in developing microorganism assemblages for bioremediation (Classen et al. 2015). In this phenomenon, plausible explanation could be attributed to the ability of chemicals to exert evolutionary pressure allowing only the species of microorganism to survive under the lethal doses and extreme environmental conditions. Therefore, exploring more promising microorganisms for consortium build up to develop resilient microbial activities in agro-farming system is vital to optimize ‘clean up’. In this study, we therefore sought to isolate and characterize organophosphate degrading bacterial strains to optimize on soil health.

## Results

### Effects of diazinon on bacterial population dynamics

Results on population dynamics reveals that, increase in diazinon concentration repressed bacteria colony count across days after incubation. The colony count differed significantly among the experimental diazinon concentrations. In the first day after incubation, diazinon at the concentration of 50 and 100 ppm decreased the number of culturable bacterial cells by 50% and 73%, while in the second day, the colony count was reduced by 47% and 70% respectively. Similar results were observed 10 days after incubation. The findings of the present study further show that, in all the treatments, the greatest decline in bacterial colony was observed in the control followed by 50 ppm treatment. On average, over 2 × 10^8^ CFU mL^−1^ bacterial colonies were observed on the third day, followed by a sharp decline in bacterial count by 10th day after incubation. Slight decline in population count from 9.33 × 10^6^ CFU mL^− **1**^ to 8.78 × 10^6^ CFU mL^−1^ in soil treated with diazinon at 100 ppm concentration 10 days after incubation was evident (Table 1).

**Table 1:** Effect of diazinon on bacterial count (× 10^6^ CFU mL^−1^)

Treatment	Count (× 10^**6**^ CFU mL^**−1**^)		
	Day 1	Day 2	Day 10
0 ppm	21.00^a^	31.00^a^	23.22^a^
50 ppm	10.67^b^	16.33^b^	13.86^b^
100 ppm	5.67^c^	9.33^c^	8.78^c^
HSD_0.05_	4.85	3.318	4.97
CV%	11.08	13.39	11.91

Numbers followed by same letters indicate the treatments do not differ significantly

### Isolation of diazinon degrading microorganisms

**Fig. 1:**
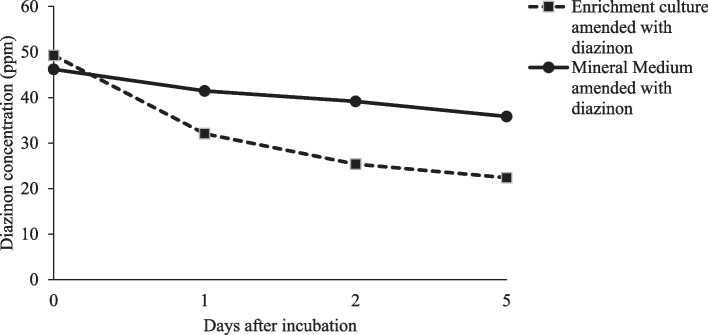
Effects of diazinon degradation by soil microorganisms’ in enrichment culture from day 1 to 5 after incubation. Mineral medium amended with diazinon was used as control

Following successes in diazinon degradation (Fig. 1) in the enrichment culture, a total of 46 strains were isolated. It was observed that, diazinon residue reduction was higher within the first 24 hours of exposure to soil microorganisms than the succeeding period. From the current study, only three isolates herein referred as S6, S36 and S37 amongst the 46 strains isolated from the agricultural field, were found effective and promising in degrading diazinon. Therefore, strains were used for further studies.

### Diazinon degradation using isolated bacterial strains in mineral medium and soil aliquot

**Fig. 2:**
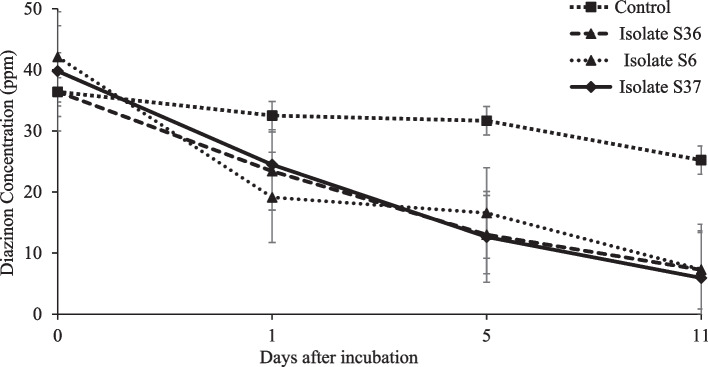
Effects of diazinon degradation by isolate S6, S36 and S37 in medium mineral compared with a control (diazinon at 50 ppm concentration dissolved in mineral medium). Standard error bars overlap indicates the treatments were not significantly differently

**Fig. 3:**
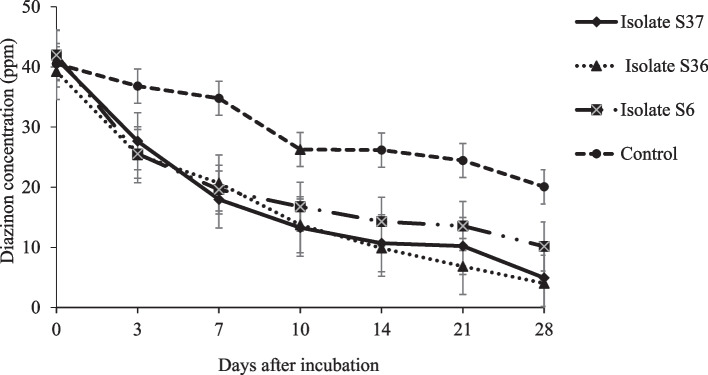
Effects of diazinon degradation in soil aliquot amended with diazinon (50 ppm) by isolate S6, S36 and S37 compared with control (Diazinon at 50 ppm concentration dissolved in soil aliquot). Standard error bars overlap indicates the treatments were not significantly differently

On average, the strains S6, S36 and S37 showed significant diazinon degradation of 49.5, 49.7 and 49.9% respectively from 1st to 11th day after incubation in the mineral medium. Degradation of diazinon was not significantly different among the isolates across the days after incubation. However, the strains depicted a varying degradation aptitude. Isolate S6 showed the highest diazinon loss (51.8%) after 24 hours but followed by decelerated rate thereafter. On the other hand, isolate S36 and S37 showed constant degradation rate of 39.8 and 39.9% respectively in the mineral medium (Fig. 2). To test efficacy of diazinon degradation in soil environment using the effective strains, an experiment was conducted in soil aliquot. Observed diazinon degradation results, followed similar trajectory as in the mineral medium (Fig. 3). On average, about 75.0% of diazinon residue was degraded from soil aliquot by the isolates discretely. The findings depicts a constant diazinon degradation rate of 45.2% by the isolates from day 0 to 7th after incubation, followed by a declined rate of 20.3% after incubating for 10 days in soil aliquot.

### Degradation kinetics of diazinon by isolated bacterial strains

**Fig. 4:**
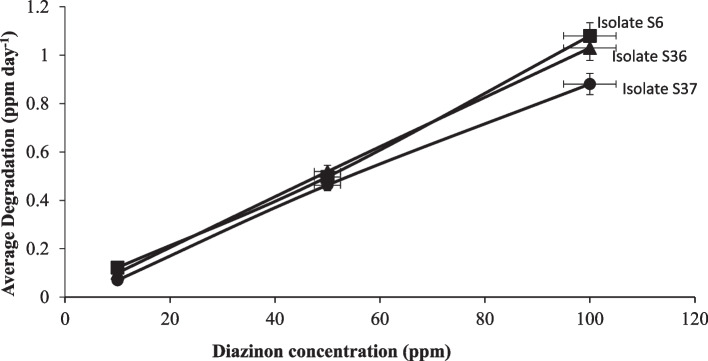
Average rate of degradation (ppm day^−1^) across diazinon concentration (ppm) associated with isolates S6, S36 and S37

**Fig. 5:**
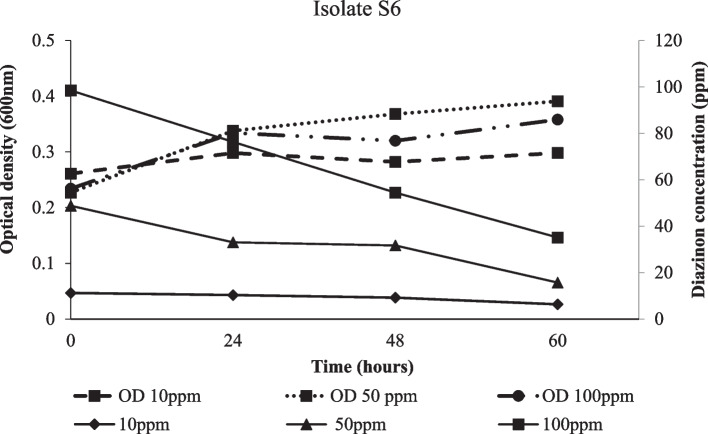
Diazinon residue (ppm) loss and isolate S6 growth in medium treated with 10, 50 and 100 ppm diazinon concentration across hours of incubation

**Fig. 6:**
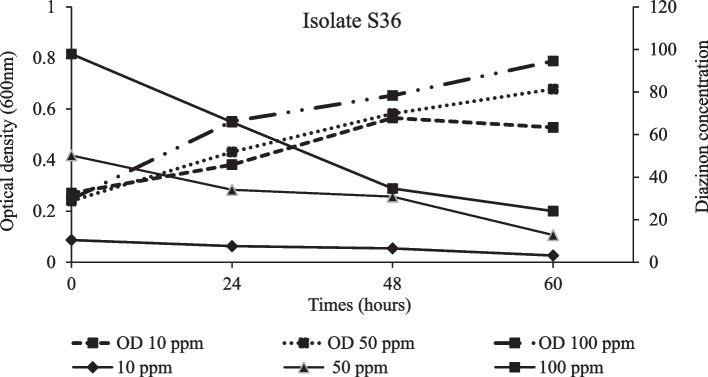
Diazinon residue (ppm) loss and isolate S36 growth in medium treated with 10, 50 and 100 ppm concentration across hours of incubation

**Fig. 7:**
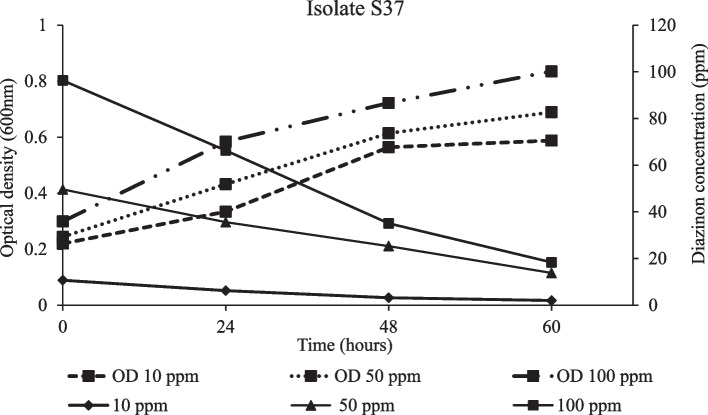
Diazinon residue (ppm) loss and isolate S37 growth in medium treated with 10, 50 and 100 ppm concentration across hours of incubation

Degradation of diazinon and bacterial strain growth was apparent in all treatments. After 24 hours of incubation, degradation kinetics attributed to the three isolates were not significantly different (*p* ≤ 0.05) at lower diazinon concentration (50 ppm). After 48 hours, isolates S36 and S37 were more effective in degrading diazinon than isolate S6. Similarly, the two isolates were not significantly different and achieved the highest degradation percentage of 74% after 60 hours. However, the highest diazinon loss was observed within 48 hours (Table 2). Parallel batch experiment showed an increase in the average rate of degradation per day following increase in diazinon concentration from 10 ppm to 100 ppm. Above 50 ppm concentration, isolates S36 and S37 showed a higher average rate of degradation per day than S6 (Fig. 4). The Highest growth rate was exhibited by isolates S36 and S37 exposed in 100 ppm diazinon concentration treatment. Furthermore, the strain S6 reached stationery growth phase 24 hours earlier than the other isolates (Fig. 5), while, a more vigorous growth following increase diazinon concentrations was observed in isolate S36 (Fig. 6) and S37 (Fig. 7). At high optical density of 0.4, 0.8 and 0.84, higher degradation rate of diazinon was evident.

**Table 2:** Rate of degradation percentage associated with isolates S6, S36 and S37 observed between 24 to 60 hours after incubation

Hours after incubation	Isolates	Rate of degradation (%)		
		10 ppm	50 ppm	100 ppm
24	Control	3.15 ± 1.20^a^	6.48 ± 1.05^a^	5.25 ± 1.65^a^
	S6	12.80 ± 3.48^b^	26.14 ± 3.09^b^	18.27 ± 2.97^b^
	S36	17.15 ± 5.42^b^	29.04 ± 2.08^b^	30.06 ± 3.63^c^
	S37	15.83 ± 7.92^b^	28.63 ± 2.00^b^	28.49 ± 3.99^c^
48	Control	5.84 ± 1.50^a^	14.79 ± 1.80^a^	16.25 ± 1.86^a^
	S6	14.41 ± 2.09^b^	28.83 ± 3.12^b^	41.82 ± 1.50^b^
	S36	27.31 ± 5.76^c^	36.36 ± 1.86^c^	58.50 ± 3.08^c^
	S37	28.93 ± 2.56^c^	36.34 ± 1.38^c^	56.82 ± 3.38^c^
60	Control	2.41 ± 1.62^a^	23.63 ± 1.24^a^	25.64 ± 1.53^a^
	S6	23.51 ± 3.51^b^	52.67 ± 3.83^b^	62.45 ± 1.09^b^
	S36	60.06 ± 4.87^c^	63.89 ± 5.41^c^	74.61 ± 1.58^c^
	S37	62.89 ± 3.51^c^	64.54 ± 5.24^c^	74.92 ± 2.65^c^
CV%		14.69		

Numbers followed by same letters indicate the treatments do not differ significantly

### Morphology and biochemical characterization of isolates S36 and S37

**Fig. 8:**
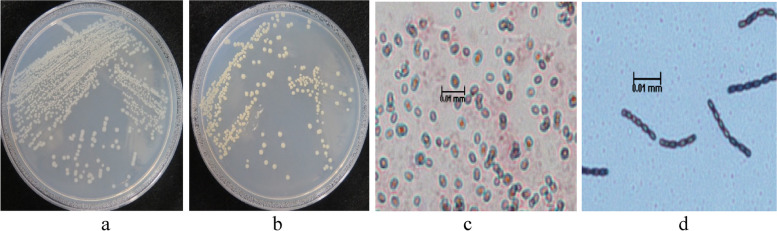
Colony colour of isolate S36 (**a**) and S37 (**b**) and Gram stain characteristics of isolate S36 (**c**) and S37 (**d**)

Following the higher efficacious ability and resource at hand, isolates S36 and S37 were selected for characterization using morphometric analysis and biochemical techniques. Morphological results following microscopy suggests that, isolate S36 was creamy white and Gram variable, while S37 was creamy yellow and Gram positive. Both isolates were rod shaped, had raised colony and formed paired chain (Fig. 8). Both isolates were able to liquefy gelatin and undergo a spectrum of reactions following 20 NE API tests, revealed the results illustrated in Table 3; while carbohydrate pattern utilization test using API 50 CH are presented in Table 4. The three isolates had distinct difference in carbohydrates fermentation and reactions. Results from API 20 NE and 50 CH tests showed that, isolate S36 and S37 were similar in most reactions by 86% and 80% respectively. Isolate S36 could not utilize D- mannose and D-adipic acid. Isolate S36 was showed urease positive, while S37 responded negatively on the test. Additionally, all the strains exhibited oxidase negative and catalase positive reactions.

**Table 3:** Reactions/enzymes as obtained from API 20 NE for isolate S36 and S37

TESTS	REACTIONS/ENZYMES	STRAINS		TESTS	REACTIONS/ENZYMES	STRAINS	
		S36	S37			S36	S37
1. Nitrate	Reduction of nitrate to nitrates	–	–	12. D-mannitol	Assimilation	+	+
	Reduction of nitrates to nitrogen	+	–	13.N-acetyl-glucosamine	Assimilation	+	+
2. Tryptophan	Indole production (Tryptophane)	–	–	14. D- maltose	Assimilation	+	+
3. D- glucose	Fermentation	–	–	15.Potassium gluconate	Assimilation	+	+
4. L- Arginine	Arginine dihydrolase	–	–	16. Capric acid	Assimilation	–	–
5. Urea	Urease	–	–	17. Adipic acid	Assimilation	–	W
6. Esculin ferric citrate	Hydrolysis (β-glucosidase)	+	+	18. Malic acid	Assimilation	+	+
7. Gelatine (bovine origin)	hydrolysis	+	+	19. Trisodium citrate	Assimilation	+	+
8.4-nitrophenyl-β D-galactopyranoside	β-glucosidase (para-Nitrophenyl- D-galactopyranoside)	+	+	20. Phenylacetic acid	Assimilation	–	–
9. D-glucose	Assimilation	+	+	21. Oxidase		–	–
10. L-arabinose	Assimilation	+	+	22. Catalase		+	+
11. D-mannose	Assimilation	–	+				

(+), (−) and (W) represents negative, positive and weak reactions respectively

**Table 4:** List of carbohydrates fermented by isolate S36 and S37

Carbohydrate	Isolate S36	Isolate S37
Control	–	–
1. Glycerol	+	+
2. Erythritol	–	w
3. D- Arabinose	–	+
4. L-Arabinose	+	+
5. D-Ribose	+	+
6. D-Xylose	–	+
7. L-Xylose	–	–
8. D- Adonitol	–	+
9. Methyl-β D- Xylopyranoside	–	W
10. D-Galactose	+	+
11. D-Glucose	+	+
12. D-Fructose	+	+
13. D-Mannose	W	W
14. L-Sorbose	–	W
15. L-Rhamnose	–	W
16. Dulcitol	W	W
17. Inositol	+	+
18. D-Mannitol	+	+
19. D-Sorbitol	–	W
20. Methy-α D-mannopyranoside	–	W
21. Methy-α D-Glucopyranoside	+	W
22. N-acetyglucosamine	+	+
23. Amygdalin	+	+
24. Arbutin	+	+
25. Esculin ferric citrate	+	+
26. Salicin	+	+
27.D-Celiobiose	+	+
28. D-Maltose	+	+
29. D-Lactose	+	+
30.D-Melibiose	+	+
31. D-Saccharose	+	+
32. D-Trehalose	+	+
	+	+
33. Inulin	W	W
34. D-Melezitose	+	W
35. D-Raffinose	+	+
36. Amidon	+	+
37. Glycogen	+	+
38. Xylitol	W	W
39. Gentiobiose	+	+
40. D-Turanose	+	+
41. D-lyxose	W	W
42. D-Tagatose	–	W
43. D-Fucose	W	W
44. L-Fucose	W	W
45. D-Arabitol	W	W
46. L-Arabitol	–	W
47. Potassium gluconate	+	+
48. Potassium 2-ketogluconate	+	+
49. Potassium 5-ketogluconate	+	+

(w), (+) and (−) represents weakly, positive and negative reaction respectively

### Molecular characterization of the diazinon degrading bacteria

**Fig. 9:**
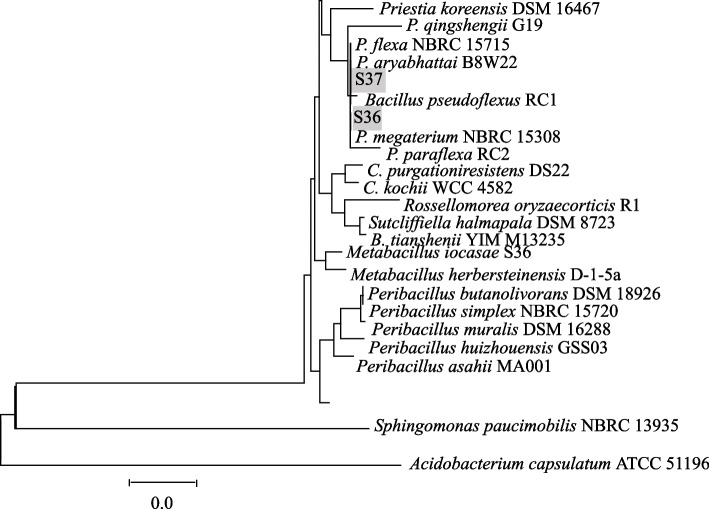
Phylogenetic position of isolates S36 and S37 based on comparative analysis of 16S rRNA genes nucleotide sequences. The scale corresponds to 2 nucleotide substitution per every 1000 nucleotides (evolution distance)

Conferring to 16S rRNA gene-based phylogenetic tree, S36 and S37 were similar in most instances and belonged to Bacceliaceae, genus *Priestia* previously named *Bacillus*. The isolate S36 and S37 showed the highest sequence similarity of 99.6% and 99.2% with type of strain of species *P. megaterium* and *P. arybhattia* respectively. Subsequently, phylogenetic tree constructed with neighbour-joining group showed that isolate S36 and S37 were similar to *Priestia* species (Fig. 9). The closeness results are in tandem with the findings of biochemical characteristics.

### Physiological studies

**Fig. 10:**
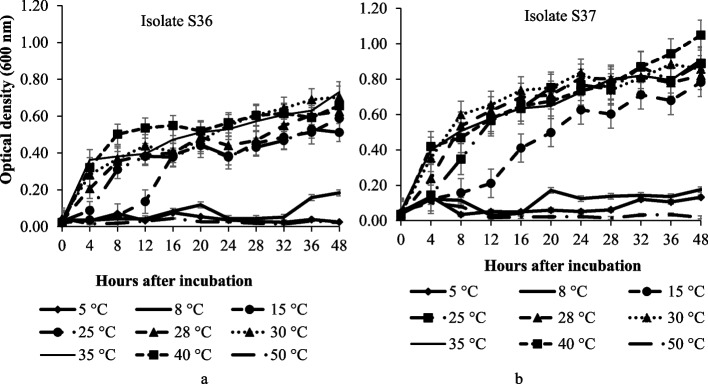
Effects of temperature (°C) against time after incubation on isolate S36 (**a**) and S37 (**b**) growth

**Fig. 11:**
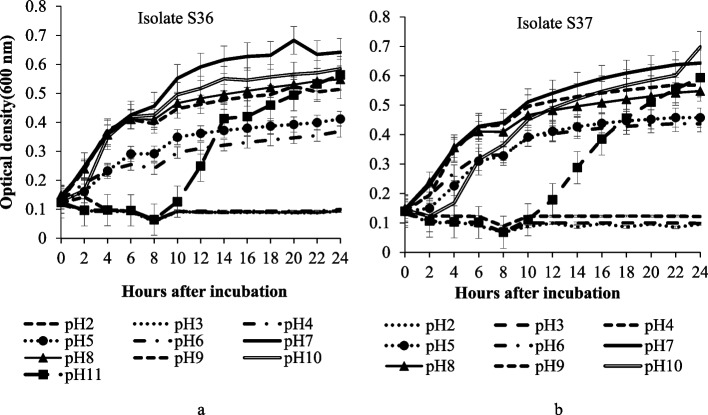
Effects of pH of isolate S36 (**a**) and S37 (**b**) across hours after incubation

Isolates S36 and S37 were screened on temperature and pH regimes to establish favourable growth conditions as well as evaluate tolerance to extreme conditions. Our results suggests that, temperature and pH had significant effect on the growth of the bacterial strains. Based on growth curtailing profile, extreme temperatures were 5°C, 8°C and 50°C while optimal temperatures were observed in the range of 25°C to 40°C in both isolates. Isolate S37 showed higher growth rate under similar temperature condition than S36 and therefore seemed more stable within 48 hours. Congruently, the highest growth for isolate S36 (optical density of 0.7) was observed at 28°C while isolate S37 had a higher optical density of 1.2 at 40°C (Fig. 10). Concurrently, influence of pH on isolates growth was evident 2 hours after incubation. Maximum growth was observed in pH 7, in which the isolates S36 and S37 had optical density of 0.7 and 0.6 respectively. Favourable pH condition for growth was in the range of 8 to 10 in both isolates (neutral to alkali conditions). At pH below 4, both isolates’ growth was suppressed. At pH 5 and 6, there was no growth of new cells 10 hours after incubation Fig. 11.

### Effects of isolates combination on diazinon degradation in broth medium and soil

**Fig. 12:**
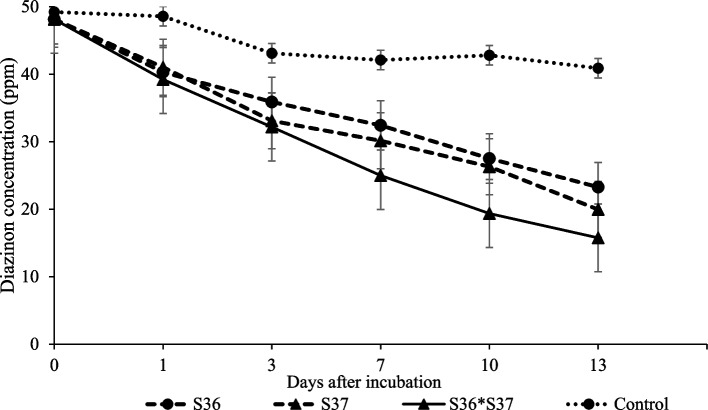
Effects of isolates S36 and S37 and their consortium on diazinon degradation from day 1 to 13 after incubation in mineral medium. Standard error bars overlap indicates the treatments were not significantly differently

From broth medium experiments, overall results showed that combination of the two isolates provided a stronger degradation in the first 10 days after incubation. Remarkably, the isolates S36 and S37 showed a degradation of 47.9 and 58.4% respectively 13 days after incubations, whereas, the isolates combination degraded diazinon by 69.2%. A higher degradation gradient was observed in combined isolate treatment than individual strains. However, the combination of S36 and S37 did not differ significantly from degradation showed by S37 after 13 days of incubation (Fig. 12). In soil medium combination of the two isolates, efficiently degraded diazinon by 69.7% as compared to medium inoculated with single strain, S36 and S37 that yielded 66.3 and 64.0% respectively, 20 days after incubation (Table 5).

**Table 5:** Effects of isolates S36 and S37 and their combinations on diazinon degradation from day 1 to 27 after incubation in soil

Isolate(s)	Diazinon degradation rate(%) across days after incubation						
	1	3	7	10	13	20	27
Control	2.57^a^	6.59^a^	16.55^a^	36.74^a^	29.26^a^	30.42^a^	33.16^a^
S36	11.77^ab^	42.16^b^	43.26^b^	57.30^b^	63.03^b^	66.32^c^	70.99^c^
S37	24.08^b^	43.64^b^	47.11^b^	57.29^b^	62.08^b^	64.04^bc^	72.90^c^
S36*S37	31.66^c^	42.76^b^	47.11^b^	57.70^b^	61.24^b^	67.97^cd^	72.21^c^
HSD(0.05)	15.81	18.55	11.985	6.10	4.55	4.95	4.61
CV%	25.22	18.26	10.18	3.89	2.68	2.82	2.39

### Degradation of other organophosphates

**Fig. 13:**
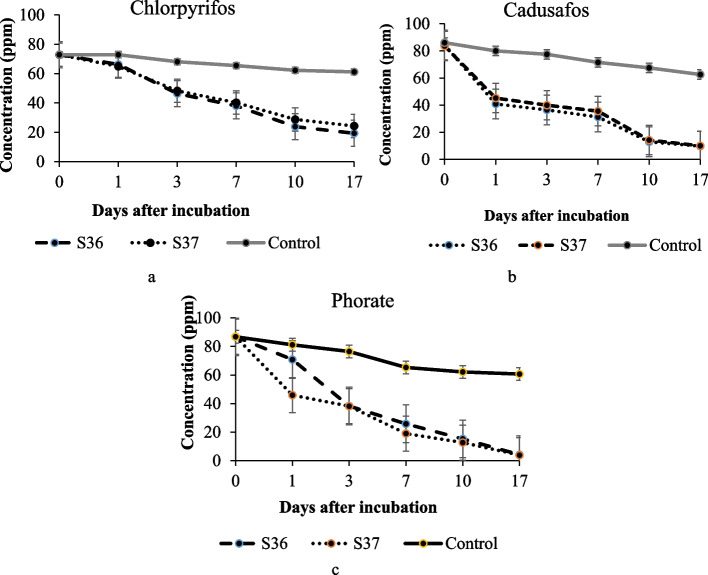
Effects of isolates S36 and S37 on chlorpyrifos (**a**), cadusafos (**b**), and phorate (**c**) degradation within 17 days of incubation

Organophosphate degradation dynamics by the isolates are shown in Fig. 13. Results obtained suggest that all isolates significantly degraded the organophosphates. Phorate was the most highly degraded (94%) followed by cadusafos (89%) then chlorpyrifos (75%) after 17 days of incubation by the two isolates. There was no significant differences in organophosphates degradation potential from day 1 to 17th after incubation among the bacterial strains. Isolate S36 degraded chlorpyrifos, cadusafos and phorate by 72%, 88% and 95% respectively while isolate S37 reduced the chemicals by 64%, 88% and 95 respectively.

## Discussion

Globally, organophosphates group is among the most widely used pesticides and often associated with irreparable consequences to human and environment. Consequently, the chemicals have attracted a considerable interests in the development of bioremediation technologies (Kaushal et al. 2021). As such, a number of pesticides degrading microorganisms have been isolated and evaluated globally to revitalize contaminated soil under different terrestrial ecosystems to restore the natural self-purifying and self-repair. Previous studies have shown that, the efficacy of the isolate varies with environmental conditions and concentration of the chemical which may limit the number of stable microorganisms capable of degrading the pesticides (Zhang et al. 2020). However, only a fraction of isolated bacterial species have been explored on and proofed effective in degrading pesticides in soil environments (Martı et al. 2004). This study therefore, endeavored to explore for bacterial species with higher degrading ability and capable of constructing a microbial consortium.

In the present study, effects of organophosphate toxicities on microorganisms’ population was evident. Increase in diazinon concentration repressed bacteria colony count across days after incubation. The decline in viable count could be attributed to the effects of diazinon toxicity on the microorganisms. Even though, there is limited literature on effects of diazinon in suppressing microbial populace, Ingram et al. (2005) and Fenlon et al. (2011) reported that, the pesticide influence urease enzyme activities of the soil dwelling organisms and mineralization process respectively. Similarly, Storck et al. (2018) found that, chlorpyrifos which has similar structure as diazinon, restrained growth of bacterial community in soil. The sharp decline in bacterial count observed from third day to 10th day after incubation could be attributed to the increased competition among the bacteria isolates over the declining resources including waning nutrients in the soil. Also, the slight decline in population count in soil amended with diazinon at 100 ppm concentration after 10 days of incubation, suggests that, diazinon was source of nutrition for the microorganisms. Our findings results of this study on effects of diazinon on microorganisms are in agreement with Wang et al. (2019a, 2019b) and (Muturi et al. 2017) report. The reduction in population could be suggesting that, certain microorganisms preferred the pesticide as source of nutrition which decreased with an increase with population. This is in support with previous studies which also hypothesized that, microbes are discerning in the source of carbon (Schrader and Blevins 2001).

Diazinon concentration was reduced by 72% by the end of 7th day in the enrichment culture in which the 46 bacterial strains were isolated. The decline in diazinon toxicity levels in the enrichment culture, further provided an insight on possibility of the presence of microbial strains utilizing the pesticide as source of food. In support of this, research findings have showed that, nutritionally, nitrogen and carbon are among the most important minerals extracted by microbes, essential for their growth and energy (Nisha et al. 2018). Correspondingly, diazinon structure provided sufficient and readily available carbons to microbes upon mineralization. The successes of the microbes to solubilize diazinon, is based on the possession of diverse mechanisms to mineralize the xenobiotic to plummet the residue levels in soil (Thabit and EL-Naggar 2013). Certainly, Drufovka et al. (2008) and Nasrollahi et al. (2020) observed that, bacteria could utilize carbon from pesticide source often reducing the chemical concentration. Our findings supported the hypothesized view that, microbial species differ in their remediation bioactivity on toxins.

Degradation kinetics showed the relationship between exponential growth phase of the bacterial cells, the period in which higher amount of energy and diazinon concentration. This relationship was also reported by Martin et al. (2003). Even though, previous studies suggested a degradation efficiency of up to 90% provided by *Pseudomonas* (Essa et al. 2016) and *Priesti*a bacteria (Pailan et al. 2015) after 21 days, 74% degradation achieved within 60 hours, connotes possibility of prominent degradation in the long run. Higher growth rate exhibited by isolates S36 and S37 in 100 ppm diazinon concentration treatment suggested that, the strains tolerated the insecticide more than isolate S6. Higher growth rate exhibited by isolates S36 and S37 in 100 ppm diazinon concentration treatment suggested that, the strains tolerated the insecticide more than isolate S6. Its worthy noting that higher inoculum (higher multiplication rate) has potential for compensating for the loss of bacterial cells that reach stationery phase early (He et al. 2018). Significance of diazinon as source of carbon was demonstrated by a sharp decline in isolates growth in medium amended with diazinon at 10 ppm after 48 hours. This could be attributed death of bacterial cells following depletion of carbon source (Wang et al. 2019a, 2019b). Hence isolate S36 and S37 were selected for further study.

Following the evaluation of diazinon degradation ability attributed to the 46 bacterial strains isolated, a varying degradability aptitude in both soil aliquot and mineral media was portrayed. One of the key attribute of promising strain in bioremediation is the ability to have high stable growth rate under varying toxin concentration (Kebede et al. 2021) and thereby providing the best tool for optimization in bioremediation. Physiological studies showed delayed growth at 15°C within the first 20 hours. This could imply presence of adaptability attributes after exposure to shock temperatures. The trajectory could be due to the need for conducive temperature for the bacteria and their enzymes to growth and survive (Sartoros et al. 2005). Hugo et al. (2014) and Arbeli and Fuentes (2010) in their study concluded that the success of biodegradation depends on adaptability of the microbes to the contiguous temperature regimes. in addition, previous study suggest that, pH influence the rate of bacterial growth and pesticides degradation (Ali et al. 2014). Besides, thrilling pH and temperatures affect availability of nutrients which may jeopardize microbial growth (Gupta et al. 2015).

Our isolates showed responses in utilization of carbon sources and enzymatic activities and oftenly differing in merely fewer occasions. Even though, Elarabi et al. (2020) isolated closely related strain to S37, that could degrade glyphosate, our isolate (S27) showed negative oxidase reactions, but similar in catalase metabolism. Similarly, reactions on sorbital, ducitol, rhamnose, adonitol and D- xylose differed, signifying that, the two strains were diverse. On sequence analysis of 16S rRNA, the two isolates were found to belong to Bacceliaceae family, genus *Priestia* which was previously named *Bacillus*. Explicitly, isolates S36 and S37 showed the highest sequence similarity with *P. megaterium* and *P. arybhattia* respectively. In correspondence, a number of studies have showed that, bacterial strains in the genus *Pristia* are versatile in degrading pesticides including atrazine (Abd Rani et al. 2022), chlorpyrifos (Varghese et al. 2022) and benzoate (Esikova et al. 2021) using diverse metabolic pathways in mineralizing toxins and pesticides (Li et al. 2022). Moreover, Pailan et al. (2015) reported that *B. arybhattia* SanPS1 could tolerate chlorpyrifos and parathion at concentrations of 500 μg mL^−1^. Similarly, Amani et al. (2019) suggested that *Acinetobacter* bacteria degraded 88.25% of diazinon within 20 days in mineral medium, whilst, our strains could degrade the pesticide up to 75% within 14 days.

Combination of bacterial isolates provided a stronger degradation in the first 10 days after incubation in mineral media and soil aliquot than individual strains. The results were similar to findings (Liao and Xie 2008). It was found that, unexpectedly, combination of 2 isolates did not provide a stronger comparative rate of degradation as observed by Wójcik et al. (2009). In our views, this could be attributed to possibly of confounding factors including materials released by this bacterial isolates that may be antagonistic to each other thereby influencing their synergistic behaviour (Tyc et al. 2017). However, it’s inevitable that consortium presented a higher tendency to increase diazinon degradation than when single strain was applied.

Phorate was the most highly degraded (94%) followed by cadusafos (89%) then chlorpyrifos (75%) after 17 days of incubation. The results are in agreement with Gevao et al. (2000) and Esikova et al. (2021) preposition that, certain bacterial strains may have bioactivity against specific substance. These organophosphate loss phases could be matched with log phase and transition to stationery phase as previously described by Karpouzas and Walker (2000). This trend suggests that the isolates lag phase is short and proliferation of the strains is within 24 hours.

## Conclusions

Biological degradation of pesticides using microorganisms is an efficient and low cost tool useful in removing pesticides residues and toxins from soil. We therefore aimed at developing a more stable microorganism for degrading diazinon and other organophosphate. From the present study, we conclude that, a number of bacterial strains were isolated and therefore are key in bioremediation activities. Diazinon tolerant bacterial isolates were isolated from agricultural field which belonged to genus *Priestia.* The strains degraded diazinon in both broth medium and soil. Degradation kinetics results showed that pesticide degradation by microbes was evident and their potential effects differ with bacterial strain. Combining of the two bacterial isolates improved degradation of diazinon by 67%. Moreover, results suggests that, still some pesticides remain in soil at the end of experiment. This means an extended period for microbial exposure to pesticides is required.

## Materials and methods

### Soil samples and chemicals

Soil samples were randomly collected from crop field which had potato crop previously. The soil was taken at a depth of 0–20 cm and stored at 4°C in laboratory. From composite sample, 800 g was ground, mixed thoroughly and passed through 2 mm sieve. Analytical organophosphates (diazinon, cadusafos, phorate and chlorpyrifos) grade (95% purity) were utilized in this study. Other chemical reagents with high commercial purity were sourced from local market of Kenya.

### Population dynamics of soil microbes

To approximate effects of diazinon toxicity on bacterial count, an experiment was conducted using sieved soil sample (unsterilized). From the sample, 10 g were weighed and moisture adjusted to 15% of the soil moisture using deionized water. The sample was treated with diazinon at a concentration of 50 and 100 ppm and placed in 50 mL bioreactor tubes in three replicates. Control included diazinon-untreated soil. The set up was incubated at 28°C for 7 days. Gentle shaking was conducted daily manually by hands (inverting 4 times) after every 24 hours. Then, 2 g was sampled and suspended in 6 mL of mineral medium and serial diluted using saline solution (0.85% NaCl) to 10^−6^ and 10^−7^ concentration. Dropwise, 80 μL was placed at the centre of 90 mm petri dish containing R2A agar media (BD Difco, USA) and spread uniformly on the media. The plates were incubated at 28°C and data on colony count collected after 1, 2 and 10 days.

### Isolation of diazinon degrading microorganisms

Microorganisms were isolated from enrichment culture prepared by mixing 5 g of with 20 mL of mineral medium. The composition of the medium was Na_2_HPO_4_.12H_2_O (3.58 g), anhydrous KH_2_P0_4_ (1.361 g), (NH_4_)_2_SO_4_ (0.3 g), MgSO_4_ (0.05 g), CaCl_2_ (0.0058 g), trace minerals (10 mL), vitamin solution (1 mL) and deionized water (1 L) at pH 7 amended with diazinon at concentration of 50 ppm. The suspension was placed in shaker incubator at 28°C at 150 rpm in dark to prevent photodegradation of diazinon. Samples for high performance liquid chromatography (HPLC) analysis were collected after 4 hours and then after 1, 2 and 5 days of incubation. When more than 50% diazinon degradation was observed, 10% of the enrichment culture was serial diluted to 10^−6^ concentration. Soil aliquot droplet (80 μL) from the culture was spread on 90 mm diameter petri dishes containing mineral medium agar amended with diazinon (50 ppm). Colonies that differed morphologically were subsequently sub-cultured to obtain pure colonies for pesticide degradation.

### Diazinon degradation in broth medium and soil aliquot using isolated strains

Successfully isolated candidate strains here in referred as S6, S36 and S37 (standardized to optical density of 1.0 at wavelength of 600 nm using spectrophotometer (Jenway 7315 spectrophotometer, Biosan)) were suspended in sterile 0.85% NaCl solution. Mineral medium (30 mL) was amended with diazinon (50 ppm) and was inoculated separately with each bacterial suspension. Additionally, soil aliquots (10 g of autoclaved soil amended with 20 mL mineral medium) were also inoculated separately with bacterial strains to test the diazinon degradation under soil based environment. Control included diazinon-uninoculated media. The set up was incubated at 28°C and residues analyzed after 4 hours and then 1, 3, 7, 10 and 11 days after incubation for broth medium while in soil aliquot, analysis was extended to 21 and 28 days after incubation.

### Diazinon analysis using high performance liquid chromatograph (HPLC)

Diazinon standard was purchased from Kemidas Company limited (dissolved in acetone) whose wavelength was predetermined (192 nm). Absorbance peak and retention time were preliminarily determined before conducting residue analysis. HPLC model (Shimadzu SCL- 10 Avp HPLC System) equipped with a Shimadzu SPD- 10 Avp photodiode array (PDA) detector (Shimadzu) and C18 column (Zobrax Eclipse Xdb-18, 80 Å, 4.5 × 150 mm, 5 μm, Agilent) was used. Analysis conditions were as follows: Ratio of acetonitrile:water (70:30), oven temperature (40°C), injection volume (20 μL), flow rate (0.7 mL min^−1^) and UV detector (190–300 nm).

### Degradation kinetics

To determine degradation kinetics, mineral medium broth was amended with diazinon at concentrations of 10, 50 and 100 ppm separately and isolates inoculated at concentration of 1 × 10^6^ CFU mL^−1^ separately into the mixture. Control included media without isolate. The treatments were replicated three times and incubated at 28°C in shaking incubator (150 rpm). Analysis for diazinon residue using High performance Liquid Chromatograph analysis (HPLC) (Shimadzu) and optical density at wavelength of 600 nm (OD_600_) using Spectrophotometer was taken after four, 24, 48 and 60 hours after incubation. Rate of degradation of diazinon was calculated using the formula below as described by Essa et al. (2016).

$$\textrm{r}=\frac{{\textrm{C}}_0-{\textrm{C}}_{\textrm{t}}}{C_0\left(\Delta \textrm{t}\right)}\ \times\ 100$$

Where C_0_ and C_t_ are the concentration of diazinon at t = 0 and time = t respectively while r is the rate of degradation.

### Morphological and biochemical characterization

Morphological identification of the isolates S36 and S37 was conducted by visual description and Gram staining technique as described by Becerra et al. (2016). Biochemical assays were conducted using Analytical Profile Index (API 50 CH and API 20 NE kits; bioMerieux) (Ashraf et al. 2019) following manufacturer’s protocol. To determine if the strains have oxidase and catalase enzyme, presence of oxidase enzyme was tested (colour change) using filter paper soaked in presence of oxidizing reagent and isolate suspension droplet, while catalase test (air bubbles) was conducted by placing isolate drop on a glass slide and adding hydrogen peroxide dropwise.

### Molecular and phylogenetic analysis

Molecular characterization was conducted following modified (Kumar et al. 2017) procedure. Deoxyribonucleic acid (DNA) was extracted using NucleoSpin® Microbial DNA protocol. Polymerase chain reaction (PCR) was performed using template of amplified 16S ribosomal RNA gene. The PCR primers included; 27f (5’- AGAGTTTGATCMTGGCTCAG-3’) and 1492r (5’- GGYTACCTTGTTACGACTT-3’). Subsequently, PCR mixture consisted of forward primer (1 μL) reverse primer (1 μL), 5X buffer (5 μL), Taq polymerase (0.25 μL), dNTP (1 μL), BSA (2.5 μL) and sterilized distilled water (13.25 μL). Precisely, 1.0 μL of template was added to make a total volume of 25 μL. PCR conditions were set as follows; denaturation (95°C for 5 minutes and further 1 minute) followed by 30 cycles of annealing (55°C), extension (72°C for 1 minute and 30 seconds) and elongation (72°C for 10 minutes) and then held at 8°C. Cleanup of PCR product was conducted following NucleoSpin® protocol. The PCR product bands were visualized using gel electrophoresis to confirm the amplification via Gel-Doc (Vilber Quarmat, Bioneer) where 1 kb ladder (NC™, NanoCell) was utilized. Sequencing was performed using SolGent co. Ltd. The raw sequences were aligned with reference sequences for phylogenetic dendrogram, which was constructed using neighbor-joining algorithm with bootstrap value of 1000 replicates in MEGA 8.0 software. The working reference sequences were downloaded using EzBioCloud database.

### Physiological studies

Physiological studies were performed following Ahn et al. (2018) procedure with slight modification. Isolate concentrations were standardized (OD_600_= 1) and exposed to 5, 8, 15, 25, 30, 35, 40 and 50°C temperature variations for 48 hours in five replications. Optical density was measured after every 4 hours. Tolerance to pH was conducted using R2A broth adjusted to 2, 3, 4, 5, 6, 7, 8, 9, 10 and 11 pH values and plated in bioscreen microplate reader (SpectraMax 340). Isolates S36 and S37 were then inoculated (1 × 10^6^ CFU mL^−1^).

### Diazinon degradation by isolates S36 and S37, and their consortium in broth medium and soil

The isolates and their consortium was evaluated against diazinon degradation in broth mineral medium and soil (autoclaved at 121°C for 60 minutes) separately. Efficiency of autoclaving was tested by serial diluting 5 g of the autoclaved soil and inoculating R2A media in plated petri dish. The strains’ cells were suspended in saline solution (0.85% NaCl) and standardized to 1 × 10^8^ CFU mL^−1^ and separately added as a single strain or combined into glass test tubes containing 20 mL of mineral medium amended with diazinon at 50 ppm concentration in triplicates. The set up was incubated at 28°C and samples taken after 1, 3, 7, 10, 14, 17 days of incubation for diazinon analysis. Control included non-inoculated mineral medium. Diazinon degradation in soil was performed using 500 g pre-treated with 50 ppm diazinon in acetone solution under sterile conditions, mixed and aired dried for 3 hours to evaporate acetone under flame hood. Then, 20 g was transferred to Tubespin® Bioreactor and moisture level adjusted to about 16% using deionized water. The isolates suspended in saline solution were separately inoculated into the soil to give a final concentration of 1 × 10^8^ cells g^−1^ in triplicate and mixed thoroughly using a sterilized spatula. Control tubes included soil without the isolates. Moisture was adjusted and maintained to about 16 ± 2% of water holding capacity of the soil throughout the experiment and incubated in the dark at 28°C. Diazinon residue analysis was conducted by soil sampling 2 g aseptically for residue analysis after 4 hours, and then after 3, 5, 7, 10, 14, 21, and 28 days of incubation.

### Degradation of other organophosphates by isolate S3 and S37

Degradation of organophosphates namely; chlorpyrifos, cadusafos and phorate was similarly conducted in mineral medium. The analytical pure grades were obtained from Sigma Aldrich Company while dissolved in acetonitrile and introduced to mineral medium broth as sole source of carbon to give a final concentration of 100 ppm. Isolates suspensions were standardized to 1 × 10^6^ CFU mL^−1^ and separately inoculated to the broth medium. The treatments were replicated three times. Control included medium without isolates. The set up was incubated at 28°C and residues analyzed after 4 hours and day one, three, seven, 10, 14 and 17 after incubation and then degradation rate determined.

### Statistical analysis

Data on colony count from population dynamics experiment was first transformed using log_10_ before conducting analysis of variance using SAS version 8.2. Similarly, data on diazinon concentration from combination test was conducted. Mean values and standard errors were calculated to separate means between and among treatments.

## Data Availability

The correspondence agrees to supply data whenever required by editor.

## Abbreviations

OP: Organophosphate
HPLC: High Performance Liquid Chromatography
Na_2_HPO_4_. 12H_2_O: Disodium hydrogen phosphate dodecahydrate
KH_2_PO4: Potassium dihydrogen phosphate
(NH4)_2_SO_4_: Ammonium sulphate
MgSO4: Magnesium sulphate
CaCl_2_: Calcium chloride
L: Litre
mL: milliliter
NaCl: Sodium chloride
UV: ultraviolet
Min: Minute
CFU: Cell Forming Unit
OD: Optical density
API: Analytical Profile Index
PCR: Polymerase Chain Reaction
ppm: parts per million
HSD: Honest Significant Difference
